# How Can the Microbiologist Help in Diagnosing Neonatal Sepsis?

**DOI:** 10.1155/2012/120139

**Published:** 2012-01-26

**Authors:** Michela Paolucci, Maria Paola Landini, Vittorio Sambri

**Affiliations:** Unit of Microbiology, Department of Haematology and Oncology “L. e A. Seràgnoli”, University of Bologna, Via Massarenti 9, 40138 Bologna, Italy

## Abstract

Neonatal sepsis can be classified into two subtypes depending upon whether the onset of symptoms is before 72 hours of life (early-onset neonatal sepsis—EONS) or later (late-onset neonatal sepsis—LONS). These definitions have contributed greatly to diagnosis and treatment by identifying which microorganisms are likely to be responsible for sepsis during these periods and the expected outcomes of infection. This paper focuses on the tools that microbiologist can offer to diagnose and eventually prevent neonatal sepsis. Here, we discuss the advantages and limitation of the blood culture, the actual gold standard for sepsis diagnosis. In addition, we examine the utility of molecular techniques in the diagnosis and management of neonatal sepsis.

## 1. Introduction

Neonatal sepsis is defined as a clinical syndrome of bacteremia with systemic signs and symptoms of infection in the first 4 weeks of life. When pathogenic bacteria gain access into the bloodstream, they may cause overwhelming infection without much localization (septicemia) or may be predominantly localized to the lung (pneumonia) or the meninges (meningitis).

Neonatal sepsis can be classified into two subtypes depending upon whether the onset of symptoms is before 72 hours of life (early-onset neonatal sepsis—EONS) or later (late-onset neonatal sepsis—LONS). These definitions have contributed greatly to diagnosis and treatment by identifying which microorganisms are likely to be responsible for sepsis during these periods and the expected outcomes of infection.

Common risk factors associated with the increased severity of the two syndromes are the birth weight and gestational age. As a result of differences in clinical and microbiological features for EONS and LONS, these syndromes will be discussed separately.

This paper is aimed to describe the main neonatal sepsis associated pathogens and the strategies used by microbiologist to diagnose neonatal sepsis. Data shown were obtained from a PubMed search using the following search parameters: neonatal sepsis, late-onset neonatal sepsis, early-onset neonatal sepsis, microbiological diagnosis neonatal sepsis, molecular methods, and antibiotic resistance in neonatal setting.

## 2. Early-Onset Neonatal Sepsis

Early-onset infections are caused by organisms present in the maternal genital tract. It can occur due to ascending infection following rupture of membranes or during the passage of the baby through infected birth canal and at the time of resuscitation [1]. The associated factors for EONS include low birth weight (LBW), prolonged rupture of membranes, foul smelling liquor, multiple per vaginum examinations, maternal fever, difficult or prolonged labour, and aspiration of meconium [2].

The most frequent microorganisms involved in EONS are *Streptococcus agalactiae* (GBS), *Escherichia coli*, and *Haemophilus influenzae* [3]. Very recently, a wide study by Jean-Baptiste and coworkers reported on the possible role of coagulase-negative staphylococci (CoNS) in neonatal intensive care unit (NICU), but no difference in mortality between infants with a diagnosis of definite, probable, or possible CoNS infection was observed [4].

### 2.1. GBS Early-Onset Sepsis: Prevention and Diagnosis

In the USA, GBS colonizes the genital and lower gastrointestinal tracts of 15 to 40% of pregnant women [5]. Approximately half of all neonates born to GBS-colonized women acquire surface colonization at delivery, and without intrapartum antibiotic therapy, about 1% of colonized full-term infants develop EONS. GBS disease remains the leading infectious cause of morbidity and mortality among newborns in the USA [6, 7]. The pathogenicity of GBS has been attributed to a number of virulence factors, including lipoteichoic acid, a thick polysaccharide capsule, capsular sialic acid, and the enzyme C5a-ase, which inhibits neutrophil accumulation at the site of infection [8].

Culture screening of both the vagina and rectum for GBS during late gestation in prenatal care can detect women who are likely to be colonized with GBS at the time of delivery and are consequently at higher risk of perinatal transmission of the germ [9]. Numerous studies have documented that the accuracy of prenatal screening cultures in identifying intrapartum colonization status can be enhanced by careful attention to the timing of cultures, the anatomic sites swabbed, and the precise microbiologic methods used for culture and detection of organisms [10]. Swabbing both the lower vagina and rectum (i.e., through the anal sphincter) increases the yield substantially compared with sampling the cervix or sampling the vagina without also swabbing the rectum [11]. Although swabbing both sites is recommended and the use of two swabs can be justified, both swabs should be placed in a single broth culture medium because the site of isolation is not important for clinical management and laboratory costs can thereby be minimized. Because vaginal and rectal swabs are likely to yield diverse bacteria, the use of selective enrichment broth is recommended to maximize the isolation of GBS and avoid overgrowth of other organisms.

Following enrichment, the conventional means for identifying GBS is through isolation on subculture to blood agar plates and presumptive identification by the CAMP test [12] or serologic identification using latex agglutination with group B streptococcal antisera [13]. Often, many laboratories direct inoculate the sample on a solid agar medium upon receipt of the swab in the laboratory in order to speed the identification of GBS; however, this procedure should never be used as a substitute for a selective broth medium, because as many as 50% of women who are GBS carriers have false-negative culture results [14]. More recently, chromogenic agars that undergo color change in the presence of beta-hemolytic colonies of GBS have become available [15, 16]. As with pigmented enrichment broths, these chromogenic agars can facilitate detection of beta-hemolytic GBS, but the majority will not detect nonhemolytic strains.

Great efforts in the field of GBS disease prevention are devoted to the development of a rapid, sensitive, and inexpensive test to detect GBS colonization in women who arrive at the hospital already in labor. A number of commercially developed assays for GBS antigen have been tested and proved to have high sensitivity for detecting heavy GBS vaginal colonization, but the overall sensitivity is much lower than that of selective broth culture [17, 18]. Since approximately 15% of cases of neonatal GBS sepsis occur when mothers have only light GBS colonization, immunoassays do not currently have adequate sensitivity to be clinically useful [19]. In addition more rapid techniques for identifying GBS directly from enrichment broth, or after subculture, have been developed, including DNA probes [20–23] and nucleic acid amplification tests (NAAT) such as polymerase chain reaction [24, 25]. These studies demonstrate the high sensitivity, specificity, positive, and negative predictive value of these techniques, but their implementation is limited because the enrichment phase is required.

Published studies on the performance of commercially available NAAT on nonenriched samples have demonstrated different levels of sensitivities (range: 62.5–98.5%) and specificities (range: 64.5–99.6%) when compared with the gold standard of enrichment followed by subculture [26–35]. The sensitivity of NAAT for GBS increases to 92.5–100.0% with the use of an enrichment step before testing the sample [24, 25, 35]. The use of an enrichment step lengthens the time to obtain a final result; however, for antenatal testing, the accuracy of results is much more important than timeliness.

Despite the availability of NAAT for GBS, utility of such assays in the intrapartum setting remains limited. Although a highly sensitive and specific test with rapid turnaround time could be used to assess intrapartum GBS colonization and therefore obviate the need for antenatal screening, data on currently available assays do not support their use in replacement of antenatal culture or risk-based assessment of women with unknown GBS status on admission for labor. The additional time required for enrichment of samples makes it not feasible for intrapartum testing, and the sensitivity of assays in the absence of enrichment is not adequate in comparison to culture. In addition, concerns remain regarding real-world turnaround time, test complexity, availability of testing at all times, staffing requirements, and costs. In settings that can perform NAAT, such tests might prove useful for the limited circumstance of a woman at term with unknown colonization status and no other risk factors. Even optimal NAAT would have drawbacks in the intrapartum setting, including a delay in administration of antibiotics while waiting for the result and no antimicrobial susceptibility testing for penicillin-allergic women [36]. Other rapid tests in addition to NAAT have been developed to detect GBS rapidly from nonenriched samples, including optical immunoassays and enzyme immunoassays; however, none is sufficiently sensitive when used on a direct specimen to detect GBS colonization reliably in the intrapartum setting [17, 27, 37–39].

When perinatal GBS screening is not performed and intrapartum antibiotic prophylaxis is not administered, newborns can develop systemic signs and symptoms of infection.

Cases of early-onset GBS sepsis continue to occur despite routine screening for GBS and incur significant morbidity and mortality. Microbiological diagnosis of neonatal sepsis is traditionally performed with blood cultures (BC), and despite its function as a gold standard, it suffers from the disadvantages of low sensitivity and reporting delay of 24–72 h. The diagnostic capabilities of blood culture systems have improved over the last decade with the advent of automated continuous blood culture monitoring systems. Although these systems can save time, subcultures are required for specific biochemical or other assays, ultimately needed for pathogen identification. In addition, neonatal blood cultures present unique problems with regard to reliability. Fastidious organisms, maternal antibiotic treatment, and small specimen volumes decrease the sensitivity of blood cultures. Furthermore, contamination of BC by skin microbiota such as coagulase-negative staphylococci (CONS) may be problematic. Inadequate sample volume is a frequent problem in children and neonates, and the sensitivity of blood culture improves with increased blood volume [40–42]. In neonates, where low-grade bacteremia is common (<4 colony-forming units/mL), at least 1 mL is necessary for acceptable sensitivity and specificity of BC testing [43].

To overcome the BC limitations, rapid assays have been developed to expedite detection of GBS in newborn urine and blood and facilitate initiation of specific antibiotic therapy. These tests include the rapid detection of GBS antigen with latex agglutination or DNA finding with amplification techniques. Many studies evaluated the utility of latex particle agglutination testing for antigen in the diagnosis of GBS sepsis in newborns. All authors demonstrated that sensitivity of these tests varies significantly among the commercially available assays for the detection of GBS antigenuria when concentrated and unconcentrated urine specimens were tested. These differences in sensitivity may affect the abilities of clinicians to accurately diagnose GBS sepsis before culture results are available [44–48].

In our review of the published literature, we found only one published study reporting the results of molecular techniques for direct detection of GBS DNA in newborns with suspicion of early-onset sepsis. Golden et al. evaluated a hybridization probe polymerase chain reaction (PCR) assay to detect GBS-specific *cfb* gene target DNA sequence in blood specimens [49]. Both sensitivity and specificity of the real-time PCR assay were 100%. The assay demonstrated 100% specificity when tested against 26 non-GBS bacteria. This method is capable of detecting as few as approximately 100 copies or 10 pg of GBS genomic DNA. This real-time PCR method is rapid, sensitive, and specific for the detection of GBS in neonatal blood samples and holds great promise in its utility in the diagnostic laboratory.

### 2.2. *Escherichia coli* Early-Onset Neonatal Sepsis

Intrapartum antibiotics have reduced the incidence of neonatal early-onset GBS disease. Some surveillance data suggest that this success may be at the cost of increasing rates of non-GBS infection, especially in premature neonates. Lin et al. and Stoll et al. in their recent studies [50, 51] confirmed an increase in EONS caused by *E. coli*, as noted previously [52, 53]. In addition, infants with early-onset *E. coli* sepsis show a poor outcome with high mortality and a third of the survivors manifesting neurodevelopmental impairment [54].

EONS with *E. coli *often presents at delivery and is characterized by bacteremia with or without meningitis. Septic shock due to endotoxemia may be a presenting sign. Alternatively, neonates may become colonized with *E. coli *at birth or through contact with colonized caregivers while in the NICU and may develop infection later. Environmental sources include ventilation systems and storage shelves [55]. Outbreaks of both enteropathogenic and nonenteropathogenic strains of *E. coli *have been described in the NICU [55–57].

A number of *E. coli *virulence factors have been identified and linked to neonatal sepsis, including the K1 capsule, fimbriae, hemolysin, rough lipopolysaccharide, Ibe (invasion of brain endothelium) proteins, cytotoxic necrotizing factor 1, and a cluster of genes present in pathogenic but not in avirulent strains of *E. coli *C5, a strain commonly implicated in neonatal meningitis. In a study by Friesen and Cho, *E. coli *isolates from term infants with sepsis were more likely to express multiple virulence factors than were those from preterm infants with sepsis implying that bacterial factors contribute to the infectivity of *E. coli *in term infants while host factors contribute to disease susceptibility in preterm neonates [58].

In premature infant, the bacterial epidemiology has completely changed during the last 10 years with *E. coli* becoming largely predominant, and with most *E. coli* infections being caused by strains showing ampicillin resistance [59]. Schuchat et al. demonstrated that no deaths occurred among susceptible *E. coli* infections, whereas 41% of ampicillin-resistant *E. coli* infections were fatal. Ninety-one percent of infants who developed ampicillin-resistant *E. coli* infections were preterm, and 59% of these infants were born to mothers who had received intrapartum antibiotic prophylaxis. These data suggest caution regarding the use of ampicillin instead of penicillin for GBS prophylaxis [59].

Prevention of *E. coli* sepsis, especially among preterm infants, remains a challenge. Despite the data shown on increasing incidence for *E. coli* EONS, no prevention or screening programs are possible during pregnancy and at delivery. Sepsis caused by *E. coli* can be only diagnosed on a clinical basis and with the support of BC.

### 2.3. *Haemophilus Influenzae* Early-Onset Neonatal Sepsis

*Haemophilus influenzae *may be vertically transferred from mother to infant at the time of delivery and occasionally causes EONS in preterm infants. *H. influenzae *accounted for 8.3% of EONS cases in the most recent National Institute Child Health and Human Development (NICHD) survey [52]. Most cases involve nontypable strains, with fewer than 10% caused by *H. influenzae *type b, presumably due to maternal immunity to the latter. The presentation of *H. influenzae *sepsis in preterm neonates is generally quite fulminant and often includes pneumonia simulating severe respiratory distress syndrome. Mortality has been reported as high as 90% [58].

### 2.4. *Listeria monocytogenes* Early-Onset Neonatal Sepsis

*Listeria monocytogenes* is a well-known and well-studied neonatal pathogen. An American study published in 1991 reported a rate of isolation of *L. monocytogenes* in preterm infants early-onset sepsis less than 5%, and the incidence of *L. monocytogenes *sepsis in neonates was approximately 13 per 100,000 live births in the USA as well as in Europe [60]. The vast majority of cases represent perinatal transmission, although nosocomial transmission has been reported [61, 62]. In several more recent studies, however, very low rates of *Listeria* infections have been reported [51, 63–65]. *Listeria *infection during pregnancy may result in miscarriage, stillbirth, or chorioamnionitis, often with placental abscesses [43]. Infection occurring after the fifth month of pregnancy commonly leads to premature labor and delivery, with up to 70% of cases delivering at less than 35-week gestation [66]. *Listeria *may infect the fetus through the ascending or hematogenous route, often leading to signs of severe sepsis at delivery [67]. In contrast to nearly all other organisms causing neonatal sepsis, *Listeria *is an intracellular pathogen and primarily targets cells of the monocyte-macrophage lineage. Impaired cell-mediated immunity predisposes the very-low-birth-weight (VLBW) infant to overwhelming infection with this intracellular pathogen [68].

Listeriosis should be considered if there has been maternal febrile illness or another clinical pointer. Amniotic fluid, placental tissues, maternal blood, and vaginal secretions, neonatal blood and CSF should be cultured in addition to the routine infection screening swabs.

Prenatal screening programs for *Listeria monocytogenes* colonization are not routinely performed, but it is recommended in cases of suspicion [69]. In such cases, our laboratory cultures the vaginal swabs and performs a subculture after enrichment in Tryptic Soy Broth with incubation at 4°C for 7 days. Serology is of little use because of cross-reactivity with other Gram-positive bacteria, and culture-positive patients often have no detectable antibodies [70].

## 3. Late-Onset Neonatal Sepsis

Late-onset septicemia is caused by organisms thriving in the external environment of the home or the hospital. The infection is often transmitted through the hands of the care providers. The onset of symptoms is usually delayed beyond 72 hours after birth, and the presentation is that of septicemia, pneumonia, or meningitis. The associated factors of late-onset sepsis include low birth weight, low gestational age, mechanical ventilation, total parenteral nutrition and its duration, previous antimicrobial exposure [71], lack of breastfeeding, superficial infections (pyoderma, umbilical sepsis), aspiration of feeds, disruption of skin integrity with needle pricks, and the use of intravenous fluids or central venous catheter [71]. These factors enhance the chances of entry of organisms into the bloodstream of the neonates whose immune defences are poor as compared to older children and adults. In addition, poor hand hygiene is associated with LONS and improving hand hygiene seems to be a method to prevent LONS [72].

In the most recent NICHD surveys, LONS is over 10 times more common than EONS in VLBW infants. The NICHD reported a 21% incidence of blood-culture-proven LONS among VLBW infants [73]. The incidence is higher among infants of <25-week gestation, with 46% of these infants suffering from LONS [73]. Infants who develop late-onset sepsis have a significantly prolonged hospital stay. They are significantly more likely to die than those who are uninfected, especially if they are infected with Gram-negative organisms (36%) or fungi (32%) [73].

The most frequent microorganisms involved in LONS are Coagulase-negative staphylococci (CoNS), Enterobacteriaceae, including *Escherichia coli* and *Klebsiella pneumoniae*, and *Acinetobacter baumannii *[74]. 

### 3.1. Coagulase-Negative Staphylococci Late-Onset Neonatal Sepsis

CoNS are the etiologic agents of the majority of nosocomial infections in neonates [73]. Although CoNS are common commensal organisms with little pathogenicity in immunocompetent hosts, premature neonates are particularly susceptible to invasive infection [19]. The first step in the pathogenicity of CoNS involves adherence of the bacteria to skin, mucosal surfaces, or indwelling artificial devices, such as intravascular catheters and central nervous system shunts, which are commonly used in preterm infants. Adherence of CoNS is facilitated by a capsular polysaccharide adhesin consisting of poly-*N*-succinyl glucosamine [75, 76]. Once adherence and colonization have been established, some CoNS produce an exopolysaccharide “slime,” which allows the organisms to form a biofilm and evade host defense mechanisms and antibiotic activity. The ability of CoNS to produce slime and biofilms has been linked to increased virulence in preterm infants [77]. Mixed-species biofilms of *S. epidermidis *and *Candida albicans *may be particularly pathogenic to preterm infants. A recent report demonstrated that the slime produced by *S. epidermidis *inhibited the penetration of fluconazole into mixed fungal and bacterial biofilms and, conversely, that *C. albicans *protected staphylococci from the action of vancomycin [78]. The major species involved in neonatal infection is *S. epidermidis*, which accounts for approximately 60 to 93% of CoNS bloodstream infection [77]. The majority of CoNS colonization is nosocomially acquired, predominantly from the hands of health care workers. Neonates with intravascular catheters, particularly those with central vascular catheters which remain in place for prolonged periods, are at high risk for CoNS bacteremia. In one small study, it was found that the administration of two doses of cefazolin during removal of the central venous catheter was associated with a reduction in postcatheter removal of CoNS sepsis [79]. This intervention requires further study before it can be routinely recommended.

Another significant risk factor for CoNS septicemia is the administration of intravenous lipid infusions, which provide a growth medium for the organism [80, 81]. Sepsis with CoNS is often indolent rather than fulminant, although fatalities have been reported [82].

### 3.2. Enterobacteriaceae Late-Onset Sepsis

Gram-negative enteric organisms of the Enterobacteriaceae family are common inhabitants of the neonatal intestine which may cause nosocomial sepsis. Similarly to what is described for *E. coli*, these organisms are surrounded by a capsule and fimbriae that contribute to their virulence in neonates. This capsular polysaccharide prevents activation of the alternative complement system protecting the bacteria from opsonization, phagocytosis, and bacteriolysis [83]. These organisms spread rapidly in the neonatal intensive care unit (NICU), and outbreaks with each of these pathogens have been reported in the literature. Epidemiologic studies have shown that most nursery outbreaks are due to a limited number of clones transmitted from patient to patient on the hands of health care workers. Contaminated breast milk or equipment used for breast milk expression have both been suggested as sources for Gram-negative colonisation and invasive disease [84, 85]. In addition, the transmission of nosocomial pathogens can occur through contaminated powdered infant formula, which recently caused a fatal case of *Enterobacter sakazakii *sepsis and meningitis in a preterm infant [86]. Intensive efforts at reducing nosocomial transmission of members of the Enterobacteriaceae have successfully eradicated colonization and disease with virulent enteric strains, although in some cases closing a NICU to new admissions has been deemed necessary to prevent life-threatening infections during outbreaks.

Parm et al. in their recent study, proposed a strategy to prevent LONS with Gram-negatives through the performance of surveillance cultures. Although inefficient for the prediction of Gram-negative late-onset sepsis in neonatal intensive care units, routine mucosal cultures screening for specific organisms in an outbreak (e.g., *Klebsiella* spp., *E. coli*, *Stenotrophomonas* spp. and *Pseudomonas* spp.) may offer an opportunity to improve infection control measures and enable timely initiation of appropriate antibiotic therapy [87].

### 3.3. *Acinetobacter baumannii* Late-Onset Neonatal Sepsis

*Acinetobacter baumannii* is one of the emerging causes of neonatal sepsis [88]. *A. baumannii* can persist in the hospital environment and has the ability to develop resistance to a majority of antimicrobials [89]. Carbapenem resistance is especially dangerous as almost no options are left for treatment, particularly in neonates [90]. Urgent steps need to be taken to prevent the spread of such resistant organisms in the neonatal intensive care units.

## 4. Antibiotic Resistance in Neonatal Setting

Nowadays antibiotic resistance is a widespread global problem. Methicillin-resistant staphylococci, extended *β*-lactamase (ESBL), and multidrug-resistant Gram-negative organisms represent the principal concern for clinicians who have to fight against infections. A recent study from Taiwan reported that 41% of neonates were colonized with methicillin-resistant *Staphylococcus aureus* (MRSA) in the NICU and estimated that 26% will develop invasive infections [91]. A similar infection rate of 21% was also found in a study in the USA [92]. Neonates with invasive MRSA infection have lower birth weight, are more likely to receive parenteral nutrition, or have an indwelling percutaneous central venous catheter and/or an endotracheal tube at the time of infection. Colonization with community-acquired MRSA (CA-MRSA) also emerged recently [93, 94]. CA-MRSA is acquired usually through skin contact with colonized adults, but breast milk can also be a vector [95]. Primary CA-MRSA infections are likely to be localized to skin and soft tissues, but bacteremia and life-threatening systemic infections may occur, often associated with invasive device use [96–98].

Among Gram-negative microorganism, a recent study reported that all of the *Klebsiella* and *Enterobacte*r which were the most common cause of bacterial sepsis were completely resistant to current empirical treatment protocol (ampicillin + gentamicin) [99]. These are the first-line treatment for sepsis according to the World Health Organization (WHO) recommendation. Gram-negative bacteria acquired the resistance to beta-lactam antibiotics: the prevalence of extended-spectrum-beta lactamase (ESBL) varies in different regions. India reports very high percentage of isolation of ESBL enterobacteria in different years: 87% in 2003 and 58% in 2007 [100]; in Europe the prevalence is lower, but in some regions of south Italy authors report a rate of up to 27% [101]. The risk factors for the acquisition of ESBL producing Enterobacteria were identified as birth weight, gestational age, antimicrobial treatment, and duration of hospital stay [102]. This finding confirms older publications [103–105].

## 5. Diagnosis of Neonatal Sepsis

### 5.1. Blood Cultures

As mentioned above, the isolation of microorganisms from blood is the standard method used to diagnose sepsis in the newborn infant [106]. Important procedures to improve the sensitivity and specificity of blood cultures include proper skin disinfection before collection, culturing early in the septic episode, taking an appropriate volume of blood per culture, and, if collecting through an existing intravenous device, ensuring that a peripheral culture is also collected and, where practical, more than one bottle per episode. This is not always feasible in a very tiny infant.

Despite the fact that the blood culture is the gold standard in the diagnosis of neonatal sepsis, it suffers from the disadvantages of low sensitivity and reporting delay of 24–72 h. The diagnostic capabilities of blood culture systems have improved over the last decade with the advent of automated continuous blood culture monitoring systems. Although they can save time, subcultures are required for specific biochemical or other assays, ultimately needed for pathogen identification.

### 5.2. Blood Volume

There are few clinical data on the effect of blood volume alone on blood culture outcome in newborns. In the UK, reported volumes per culture drawn vary from 0.3 mL to 0.66 mL, all well under the lower limit of 1 mL recommended by paediatric blood culture bottle manufacturers [107–109].

The issue of blood volume drawn and blood-broth dilution still remain unsolved. Evidence that supports the limited impact of the blood volume drawn comes from studies on quantitative blood cultures obtained from septic newborn in 1970s; results indicated that 80% of these patients were infected with high loads of *Escherichia coli *and since then it has traditionally been thought unnecessary to draw more than small amounts of blood [110]. Conversely, a study on infants from 2 months to 18 months of age (mean 15 months) admitted to the paediatric emergency department with suspicion of sepsis underlines the importance of increased blood volume collected. Investigators drew up to 11.5 mL blood from 300 children. Then, 10 mL was divided into one 6 mL and two 2 mL aliquots; each was further divided and inoculated into aerobic and anaerobic culture bottles. The remaining 1.5 mL was used in a quantitative culture system. In 30 significant infection episodes, the 6 mL bleed had greater detection sensitivity at 24 hours than the two 2 mL bleeds combined, and a greater final sensitivity [40]. As hypothesized by Washington II and Ilstrup, bacteraemias with high concentrations of organisms require less blood to be sampled than low density bacteraemias [111]. The concentration of a variety of common pathogens in neonatal and paediatric bacteraemias has been documented in numerous studies using quantitative culture systems [112–117]. Despite many organisms occurring in high concentrations, low-density bacteraemia is also recorded for most pathogens. In a laboratory-based study, Schelonka and colleagues [43] explored the effect of small blood culture volumes for a variety of common neonatal pathogens. A range of blood volumes containing known concentrations of neonatal pathogens were injected into standard paediatric blood culture bottles. If organisms were present at densities of <4 colony-forming units (cfu)/mL, blood volumes of 0.5 mL or less had a significantly diminished chance of detecting bacteraemia. This finding did not differ between organisms. Brown and colleagues [118], however, using similar in vitro techniques, found that placental blood seeded with more than 10 cfu/mL *E. coli *or group B streptococcus required only 0.25 mL blood to be consistently detected.

Dealing with the importance of maintenance of dilution blood broth, a prospective Mexican study supports this thesis. Authors examined infants up to 12 months with clinical signs and symptoms of sepsis. From the same venepuncture, a volume of 2.2 mL of blood was drawn from each infant and divided into 2.0 mL and 0.2 mL aliquots and injected into culture medium, maintaining a blood to total broth dilution of 10%. Compared with the 2.0 mL aliquot, the 0.2 mL sample was found to have a sensitivity of 95% and specificity of 99% for detection of significant bacteraemia [119]. These data agree with adult studies results, where dilution appeared to affect culture sensitivity. In adult setting, maximal sensitivity was reported when the blood volume was 10–20% of the total medium volume [120, 121]. Despite this evidence, Kennaugh and colleagues found no effect of dilution between 1 : 10 and 1 : 100, using 0.5 mL adult blood seeded with low concentrations of common neonatal and paediatric pathogens [122].

### 5.3. Number of Blood Cultures

There is limited information to guide the practitioner on the optimal number of blood cultures that should be obtained when evaluating an infant for suspected neonatal sepsis [106, 123–125]. Some data have suggested that, in the neonatal period, multiple site blood cultures may improve pathogen detection if bacteremia is intermittent, if there is a low density of bacteria present in the circulation, if there is an overdilution of the small volume of blood obtained during a culture with the blood culture broth, or if there is an inhibitory effect of the intrapartum antibiotic therapy administered to mothers [125]. Indeed, several authors have suggested that multiple-site blood cultures may be more efficacious in diagnosing neonatal sepsis [123, 125–127].

In adults, taking up to three blood cultures per sepsis episode increases the chance of detecting bacteraemia [111, 120, 128, 129] and the recommendations for older children and adults are to obtain blood cultures from at least two sites [125]. There are no neonatal data, as usual practice is to take only one blood culture before starting antibiotic treatment [130, 131]. This decreased sampling is attributed to the small circulating blood volumes of neonates, the potential for increased transfusion requirements, the technical difficulties posed, and the possible rapid deterioration of newborns in the setting of sepsis [132]. In children, raising the number of blood cultures to two or three bottles, whether from one or more sites, does increase yield [40, 41, 133].

To our knowledge, there is only one prospective and observational study that has been performed to determine the usefulness of two site blood cultures in the initial evaluation of neonates for sepsis. This study strongly indicated that a single site blood culture with blood volume of ≥1 mL should be sufficient to document “true” Gram positive, Gram negative, or fungal sepsis in neonates. All neonates with positive cultures had the same organism with a similar sensitivity pattern obtained from the two different peripheral sites. In this study, the blood cultures were obtained from two different peripheral sites within 15–30 min of each other in every neonate once sepsis was suspected. Since no infant grew organisms from only one of the two sites, a single site blood culture should be sufficient to document sepsis, and is biologically plausible possibly because young children have high-colony-count bacteremia [134], the bacterial clearance is slower, and the bacteremia is more continuous in neonates with sepsis than in older patients [135].

### 5.4. Timing to Blood Collection

In contrast with extensive adult data on the periodicity of bacteriemia in a variety of clinical scenarios, neonatal setting is characterized by a lack of data on timing of blood cultures. Unfortunately, the necessity to have a prompt antibiotic coverage when the infant shows signs of sepsis contributes to the lower rate of significant positive blood cultures in neonates [112].

Practically, the optimal time to culture for bacteraemia is “as early as possible” in the course of a septic episode and the interval between repeat blood cultures does not appear to be important [136]. Wherever feasible, BC should be obtained before initiation of antibiotic therapy.

### 5.5. Time to Positivity

A recent study was conducted to determine the time to positivity (TTP) of neonatal blood cultures, to investigate differences between early-onset versus late-onset sepsis, and nonproven versus proven sepsis, and to examine differences in TTP by organism type using a retrospective observational study. The TTP was recorded for all episodes of suspected sepsis in an approximately 6.5-year period. A total of 2916 blood cultures were collected, of which 437 (15%) became positive. The overall TTP was 21.33 h. The difference between the median TTP in early-onset versus late-onset sepsis was 0.83 h (22.00 versus 21.17 h, *P* = 0.75). The median TTP for Gram-negative organisms was 11.17 h, whereas the median TTP for Gram-positive organisms was 23.59 h (*P* < 0.001). In Gram-positive isolates, the median TTP for CoNS was 26.67 h, whereas the median TTP for non-CoNS was 12.83 h (*P* < 0.001). The median TTP in proven sepsis was 20.17 h, whereas it was 29.67 h (*P* < 0.001) in nonproven sepsis. TTP of neonatal blood cultures was significantly shorter for Gram-negative organisms. Authors suggest shortening the total incubation time of neonatal blood cultures to a maximum of 3 days. According to results showed, authors propose to narrow the antimicrobial spectrum to solely target Gram-positive bacteria when the culture is still negative after 48 h, and to cease antimicrobial therapy when the culture is still negative after 72 h in clinically well infants [137].

### 5.6. Results Interpretation

Interpreting positive results depends on clinical presentation, how the culture was taken, the organisms grown, and the time taken for the blood culture to become positive. Some organisms, such as *Neisseria meningitidis* and *Candida albicans*, are nearly always significant, even in the context of a well-looking child [138]. Cultures positive with potential pathogens that may also be contaminants are far more difficult to interpret, the most common of which is CoNS. These results must always be interpreted in the specific clinical context in which they are seen. Whereas CoNS grown from a previously well child presenting from the community are almost always a contaminant, CoNS growing three days later from the same child after being in hospital with an indwelling intravascular device may well be significant. There are no highly specific and sensitive criteria for determining the clinical importance of CoNS isolates based on clinical and routine microbiological parameters. Most definitions used in adults and older children involve the same organism being grown from at least two separate bleeds, not taken from indwelling intravascular devices [139]. In neonatology, usually only one blood culture is taken before the start of antibiotic treatment, making such definitions difficult to use. Rates of contamination are thought to be highest in neonates [120]. Cultures drawn through indwelling intravenous devices are more likely to be contaminated with CoNS colonising the lumen of the device, which may not be causing systemic infection. Positive blood cultures with higher colony counts and flagging positive within 48 hours of being drawn have been associated with an increased likelihood of significance, but are not absolutely sensitive or specific, and may be affected by prior antibiotic use [140–142].

### 5.7. Molecular Methods

The development of pathogen detection tools assisting blood cultures that offer more rapid results and higher sensitivity is expected in neonatal intensive care to optimize the use of antimicrobial agents. In fact, when the clinician suspects neonatal infection or sepsis, blood culture and cultures of various body sites are immediately undertaken and administration of broad-spectrum antimicrobial agents is empirically started. Because of the high risk of mortality if sepsis is left untreated, or not adequately treated, antibiotics cannot be de-escalated if cultures are still negative and the baby is though clinically to have infection. As a consequence, it is necessary to continue administration of broad-spectrum antimicrobial agents [143]. This empirical practice is associated with increased development of drug-resistant microorganisms and disturbance of intestinal flora [144, 145]. In addition, prolonged initial empirical antimicrobial therapy may be associated with increased risk of necrotizing enterocolitis or death among extremely low-birth-weight neonates [146].

Molecular methods may offer advantages due to the rapidity and the small sample volume required for analysis. These techniques, revealing the presence of microbial DNA in the sample, are based on hybridization or amplification. Technical and analytical details about these methods have been already described for adult [147] and neonatal [148] setting and are not the object of the present review. The purpose of this paper is to discuss the utility of molecular techniques in the diagnosis and management of neonatal sepsis.

Considering the wide range of bacterial species causing neonatal sepsis, a good molecular method should identify all possible pathogens and also those at low load in a short time. Unfortunately, published molecular techniques are not capable to satisfy all these properties, and the results obtained often lack sensitivity. The majority of the molecular tests described for neonatal sepsis are home made and are based on the detection of 16S rRNA gene, an ubiquitous gene that is preserved in all bacterial and comprises both conserved and variable regions [149]. The conserved regions are targeted by universal primers to identify it as a bacterium, and the variable region can be utilized in genus- or species-specific assay [40, 150].

After DNA extraction, an amplification step can be performed using different approaches: broad-range conventional PCR and multiplex PCR. The first strategy targets the panbacterial 16S rDNA region, through broad-range or universal PCR primers, and is useful when followed by sequencing or hybridization [151–153]. Multiplex PCR approach utilizes multiple primer pairs for multiple targets in a single PCR reaction, and amplicons can be detected by sequencing, hybridization, or, more often, by fluorescent probes.

These studies [145, 153–161] were conducted to analyze the analytical performance of different PCR technologies. The strength of molecular methods, as expected and underlined, is the rapidity to detect a positive or negative result. Depending on the different approach of DNA isolation, PCR employed, and detection strategy utilized, the sensitivity of these techniques varies from 41% to 100% and specificity has a range between 86% and 100% in comparison with BC. With regard to positive and negative predictive values, data shown indicate PPV ranging from 19% and 100%, whereas NPV is always higher than 90% (range: 94%–100%).

Our experience of the molecular diagnosis of neonatal sepsis is based on the employment of a commercial multiplex real-time PCR (LightCycler Septi*Fast* M^GRADE^, Roche Diagnostics). This test detects more than 25 bacterial and fungal species in a single reaction, and, to our knowledge, only two studies have published data on implementation of this test in a neonate setting [162, 163]. In one of these study, multiplex PCR was tested on 34 blood samples obtained from newborns suspected to have late-onset sepsis [162]. The second study was conducted in a paediatric hospital, and Septi*Fast* test was used to diagnose sepsis in 803 children and newborns [163]. The positivity rate for the molecular test was higher than BC, and authors speculated that the rapid detection of pathogens is important in determining the course of treatment of neonatal sepsis and that rapid confirmation of negative results is also important. Based on confirmed negative results, clinicians could promptly interrupt empiric antimicrobial therapy and avoid unnecessary antibiotic treatment in seriously compromised infants. Additionally, they can rapidly redirect the clinical investigation toward other sites or causes of infection, or even to other disease etiologies.

Examining all published data on molecular diagnosis of neonatal sepsis, it is important to highlight some critical points:

- molecular assays may improve the detection of pathogens causing sepsis; the positivity rate of PCR (range from 3% to 29%) in various studies of septic neonates is still low. The experience gained from this study illustrates the need for changes in sample collection and preparation techniques so as to improve analytical sensitivity of the assay;
- blood volume utilized for DNA isolation significantly varies among the mentioned studies (range: 200 *μ*L–2 mL), thus the lack of sensitivity could depend on the limited amount of blood extracted. By increasing the blood volume for extraction, the higher is the quantity of DNA isolated and the easier its detection. Unfortunately, technical difficulties for sample taking in newborns, especially in small, sick preterm neonates, often limit the volume of blood obtained [19];
- cases of false-negative results are reported in patients who are infected with pathogens that are not targeted in the assay;
- these assays are associated with a potential for false-positive results due to contamination from bacterial DNA, which is widespread in the environment. False-positive results can be obtained also if DNA from pathogens already killed by antibiotics is present. Molecular methods merely detect microbial “DNAaemia”;
- contrary to previous theories that PCR results are not influenced by antibiotic therapy, Dutta et al. revealed that only 12% of samples that were PCR positive prior to the start of antibiotic therapy were positive after 12 h of therapy and none were positive after 24 or 48 h following initiation of antibiotic therapy [161];
- these tests are not yet readily available in all hospitals.

In this paper, we have analyzed published data regarding the different microorganisms involved in EONS and LONS. Early-onset infections are caused by organisms prevalent in the maternal genital tract or in the delivery area. Prevention of these infections is possible through screening programs for maternal vaginorectal colonization, especially for the detection of GBS colonization in the last gestational weeks. Such programs have resulted in the reduction of GBS infections in newborns but are not currently directed to the detection of other pathogens involved in EONS. We speculate that the extension of screening programs to other pathogens might allow the administration of specific antibiotic prophylaxis. Additionally, prevention should be practiced for LONS, where pathogens are transmitted from the environment surrounding the neonate, both in hospital and at home. These practices will not reduce completely the incidence of EONS and LONS but are a strategy to control the transmission from mother to child or from environment to neonate. Improvements can also be made in the microbiological diagnosis of neonatal sepsis, especially in optimizing the gold standard culture and to cover its limitations with other rapid techniques. Molecular techniques are candidates to complement blood culture methods to diagnose neonatal sepsis, but published data underscore the cautionary stance that should be taken when considering the use of a molecular amplification test for diagnosing neonatal sepsis. Further studies are needed to define the role of molecular assays in the identification of septic infants, their impact on physician management decisions regarding antibiotics, and their effect on clinical outcome.

## 6. Conclusion

In this paper, we analyzed data available on the different microorganisms involved in EONS and LONS. Early-onset infections are caused by organisms prevalent in the maternal genital tract or in the delivery area. Prevention of these infections could be practiced through screening program for mother vaginorectal colonization. Available programs are based on the detection of GBS colonization in the last gestational weeks. These programs allow the reduction of GBS infections in newborns but are not directed to the detection of other pathogens involved in EONS. We think that the extension of the screening program to other pathogens could identify them and implement the administration of specific antibiotic prophylaxis.

On the other hand, prevention should be practiced also for LONS, where pathogens are transmitted from the environment surrounded the neonate. Thus, prevention should be actuate both in hospital and at home.

This possible practice will not reduce completely the incidence of EONS and LONS but could be a strategy to control the germs transmission from mother to child or from environment to neonate.

Improvements could also be done in the setting of microbiological diagnosis of neonatal sepsis, either to optimize the gold standard culture or to cover its limitations with other rapid techniques. 
